# Broadband nonlinear optical modulator enabled by VO_2_/V_2_O_5_ core–shell heterostructures

**DOI:** 10.1515/nanoph-2022-0142

**Published:** 2022-04-27

**Authors:** Longlong Chen, Jing Huang, Ning Li, Hao Zhu, Jianbang Hu, Lili Miao, Chujun Zhao

**Affiliations:** Key Laboratory for Micro/Nano Optoelectronic Devices of Ministry of Education & Hunan Provincial Key Laboratory of Low-Dimensional Structural Physics and Devices, School of Physics and Electronics, Hunan University, Changsha 410082, China

**Keywords:** heterostructures, nonlinear optical modulator, nonlinear optics, Z-scan

## Abstract

Broadband pulsed lasers have become an indispensable part in optical communications, biomedical engineering, materials processing, and national defense. Inspired by the broadband and ultrafast optical components, great efforts from the laser and material community have been paid to explore the emerging nonlinear optical materials. Here, we found that the VO_2_/V_2_O_5_ core–shell heterostructures with type-II staggered band alignment exhibit broadband nonlinear optical response towards mid-infrared spectral range. The nonlinear optical characterizations verify that the heterostructures show the modulation depth and saturation intensity of 27% and 42 GW/cm^2^ at 1064 nm, 23% and 78 GW/cm^2^ at 1550 nm, and 16.5% and 63.9 GW/cm^2^ at 2800 nm, respectively. With the nonlinear optical modulator, stable mode-locked Yb-doped and Er-doped fiber lasers have been realized with pulse output as short as 310 ps and 633 fs, respectively. In addition, the stable Q-switched Er-doped fluoride fiber laser has been demonstrated with a pulse repetition rate of 89 kHz and the shortest pulse width of 680 ns, respectively. The experimental results indicate that VO_2_/V_2_O_5_ core–shell heterostructures can be broadband nonlinear optical modulators from the near-infrared to the mid-infrared spectral range, offering opportunities to develop high-performance photonic devices.

## 1 Introduction

Broadband nonlinear optical materials are essential to meet the needs of ultrafast optical and photonic device applications [1], [2], [3], [4]. In a typical application, the nonlinear optical materials can operate as an intensity-dependent modulator, i.e., saturable absorber (SA), which can modulate the fiber laser system to deliver the pulsed laser via the Q-switching or mode-locking technique [5], [6], [7], [8]. To fulfill the needs of stable, broadband, and ultrafast fiber laser applications, researchers have investigated a great number of emerging materials. The first generation of SA was the dye, which was used to passively mode lock the Nd^3+^: glass lasers in 1966 [9]. After that, different nonlinear optical materials have been explored to accomplish different laser requirements. Semiconductor saturable absorber mirrors (SESAMs) are the available commercial SAs, which can satisfy the requirements towards developing high-power, high-efficiency lasers. However, the SESAMs suffer from limited operation bandwidth and complicated fabrication procedures [7]. With the discovery of graphene, different types of SAs have been investigated in different laser systems successfully [10], [11], [12], [13], [14], [15], [16], [17], [18], [19], [20], [21], [22]. However, the preparation and transfer processes inevitably lead to poor repeatability and reliability, which is challenging to find stable, broadband, and ultrafast SAs to modulate the pulsed lasers, especially towards the mid-infrared regime.

Vanadium dioxide (VO_2_) is a typical phase transition material where the temperature [23], light [24], electric fields [25], mechanical stresses [26], and electrochemistry [27] can cause an insulator-to-metal phase transition. The thermal phase transition temperature of VO_2_ is 340 K [28], and the temperature change can affect the bandgap of VO_2_, which broadens the response wavelength [29]. As a phase transition material, VO_2_ has been used in versatile optoelectronic devices, such as optical switches, sensors [30, 31], temperature actuators [32] and thermochromic smart windows [33]. The light-induced phase transition of VO_2_ can be recovered on sub-ns timescales and can be used for all-optical processors with GHz processing speeds [34]. Ji et al. have found that VO_2_ has excellent adaptive infrared camouflage in the mid-infrared wavelength range [35]. Moreover, the electrically tuned antennas [36] and tunable spectral absorption [37] in the mid-infrared waveband with VO_2_ have been achieved. Likewise, VO_2_-based metamaterials with tunable frequencies from 1.5 to 5 μm in the near-infrared and mid-infrared wavelength range have been realized [38]. With the increasing incident intensity, the VO_2_ will show an intensity-dependent nonlinear response [39, 40] to modulate the laser system. Nie et al. demonstrated a pulsed laser with a width of 700 ps in a typical Q-switched Nd: YVO_4_ waveguide cavity with VO_2_ as SA [41]. Wang et al. reported that VO_2_ nanoparticles have high optical nonlinearity, which is higher than carbon nanomaterials, gold nanocrystals, and Cu_1.8_S nanocrystals under the same test conditions. Then, they achieved broadband Q-switching operation at 1, 1.56, and 2 μm and mode-locked pulse output at 2 μm with VO_2_ as SA [42]. However, the operating wavelength of the SA has not extended above 2 μm waveband due to the intrinsic band gap limitation of VO_2_. The heterostructures with type II staggered band arrangement provide opportunities for extending the waveband of the optical response [43], [44], [45], [46], which can overcome the limits of the intrinsic band gap of the materials and exhibit excellent performance in photodetectors [46, 47], hydrogen storage [48], and solar cells [49, 50].

Here, we found that the VO_2_/V_2_O_5_ core–shell heterostructures can be evolved from VO_2_ naturally in ambient conditions. The VO_2_/V_2_O_5_ belongs to the type II heterostructure, which can extend its optical response towards the mid-infrared spectral range. With the help of the intensity-dependent nonlinearity of the VO_2_/V_2_O_5_ core–shell heterostructures, stable 1 µm and 1.5 µm mode-locked fiber lasers have been delivered successfully. In addition, the Q-switched fiber laser operating around 3 μm has been demonstrated with the VO_2_/V_2_O_5_ core–shell heterostructures. The experimental results demonstrate that the VO_2_/V_2_O_5_ core–shell heterostructures can not only broaden the operating wavelength beyond the intrinsic band gap limitation, but also avoid problems of 2D materials-based SAs, such as complicated preparation processes and low optical damage thresholds, which can offer opportunities to develop high-performance photonic devices.

## 2 Material preparation and characterization

**Figure 1:**
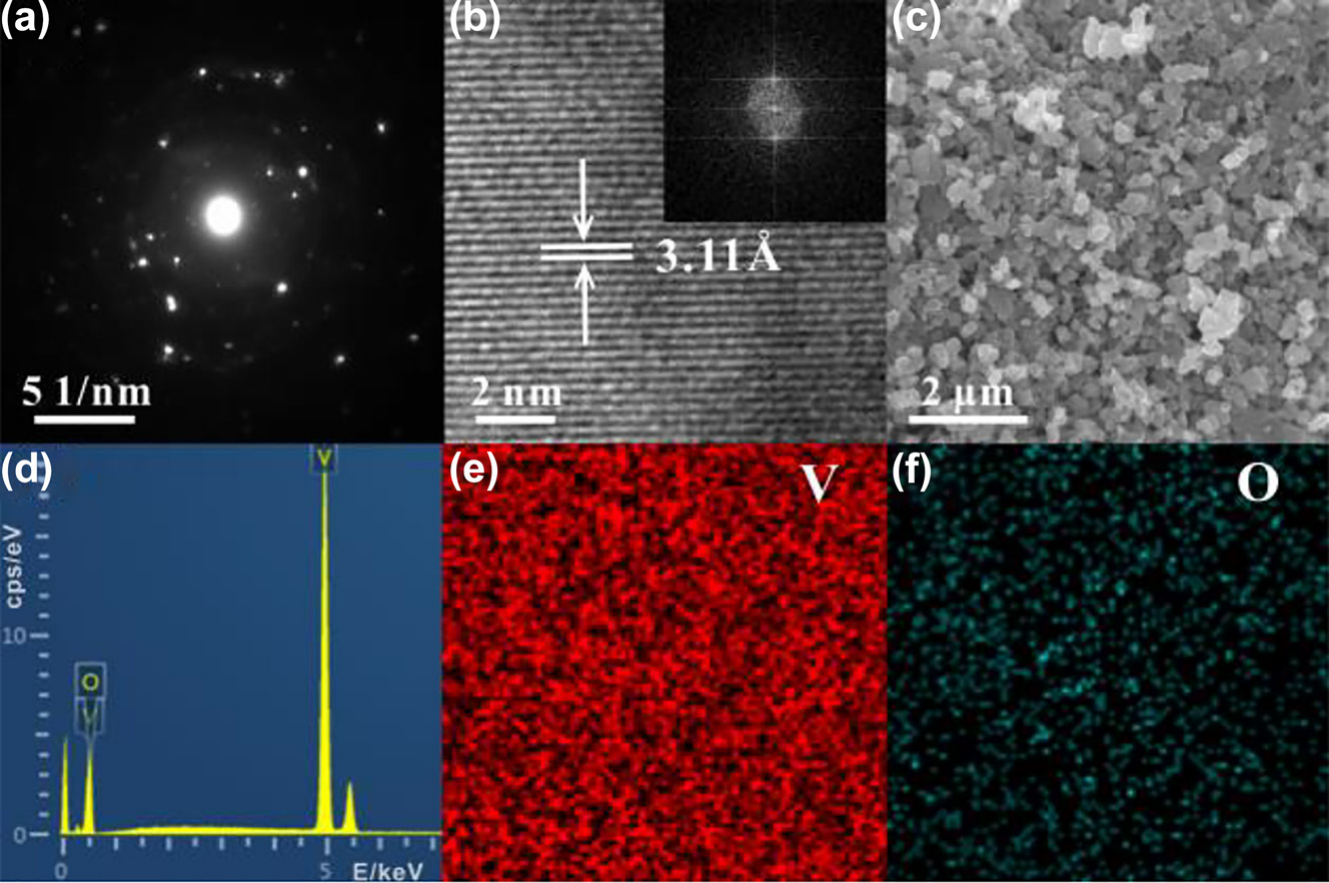
Material characterization. (a) SAED image. (b) TEM image. The inset is the FFT image. (c) SEM image. (d) EDS diagram. (e) and (f) EDS mappings of V and O.

**Figure 2:**
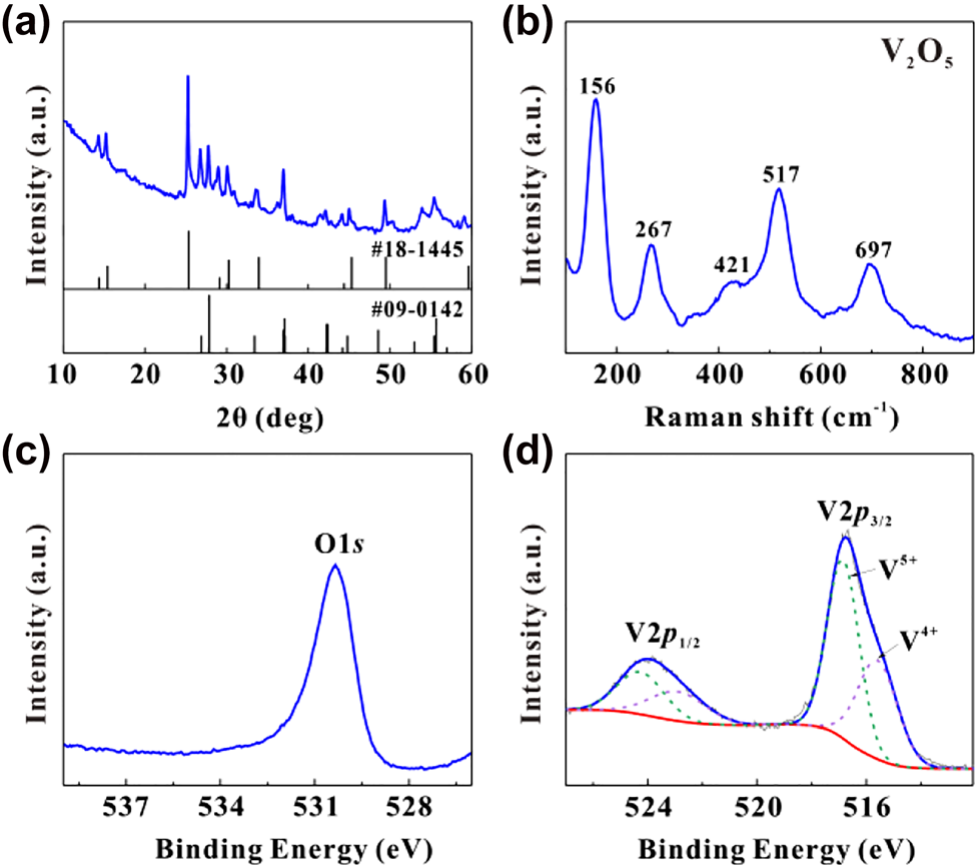
Characterization of samples. (a) XRD patterns. (b) Raman spectral lines. (c) XPS spectral lines of O element. (d) XPS peak separation spectral lines of V element.

**Figure 3:**
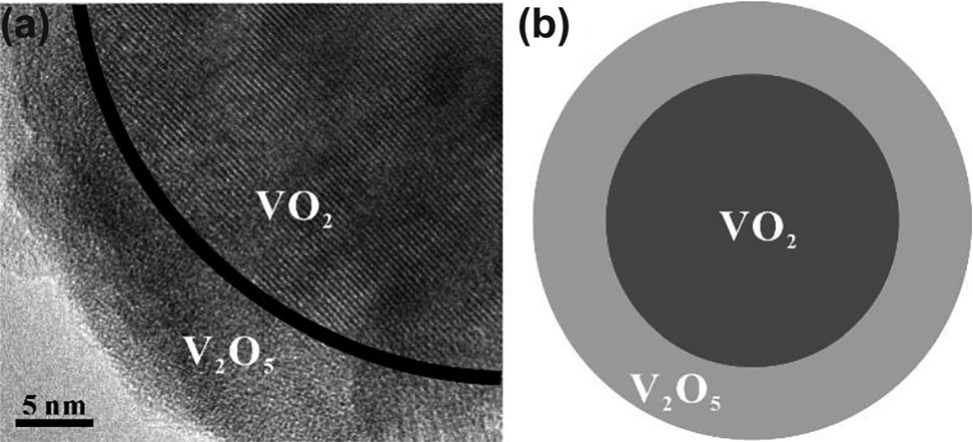
VO_2_/V_2_O_5_ core–shell heterostructures. (a) HRTEM diagram. (b) Schematic diagram of the core–shell heterostructures.

The VO_2_ particles were obtained from Macklin Biochemical Technology Co. The particles were grinded with an agate mortar for 1 h. They were subsequently left to oxidize naturally in the air for a few days. The selected area electron diffraction (SAED) result is shown in Figure 1a, from which it can be seen that the samples are polycrystalline. The transmission electron microscopy (TEM) result of the samples is shown in Figure 1b and the corresponding fast Fourier transform (FFT) is shown in the inset, where the lattice spacing of the particles is 3.11 Å. Figure 1c is the scanning electron microscope (SEM) photograph of samples, and the diagram shows that the samples characterized are generally sphere-like particles. The Energy dispersive spectroscopy (EDS) spectral line of the samples is shown in Figure 1d, and the graph shows that the samples characterized only contained elements V and O. The results of EDS mapping are shown in Figure 1e and f, and both V and O elements are uniformly distributed in the materials. The X-ray diffraction (XRD) patterns of the samples are shown in Figure 2a, and the peak positions correspond to JCPDS codes 18-1445 and 09-0142, proving that the materials used for the experiment are VO_2_. The Raman spectroscopy lines are shown in Figure 2b. The peak position of the Raman spectrum corresponds to V_2_O_5_, not VO_2_. To resolve this contradiction, a more detailed characterization of the materials has been carried out. The results of the separation of X-ray photoelectron spectroscopy (XPS) spectral lines indicate that the materials contains both V^4+^ and V^5+^, as shown in Figure 2c and d, indicating that the materials contains both VO_2_ and V_2_O_5_ [51]. The relative atomic ratio of V:O detected by XPS is 18.7:53.0, which tends to be 2:5. And the relative atomic ratio of V:O detected by SEM is 35.37:64.63, which tends to be 1:2. Since XPS is used to characterize the surface of the materials, it is known that the surface composition of the materials is mainly V_2_O_5_. XRD and SEM are used to characterize the main components of materials, the XRD and the relative atomic ratio of V:O detected by SEM results indicate that the main component of the materials is VO_2_. The high-resolution transmission electron microscopy (HRTEM) diagram of the materials is shown in Figure 3. The interior of the materials has distinct lattice fringes, while the surface of the materials has no distinct lattice fringes, as shown in Figure 3a. By comparing the previous measurement results, it is confirmed that the materials are the VO_2_/V_2_O_5_ core–shell heterostructures with the structure in Figure 3b [52].

**Figure 4:**
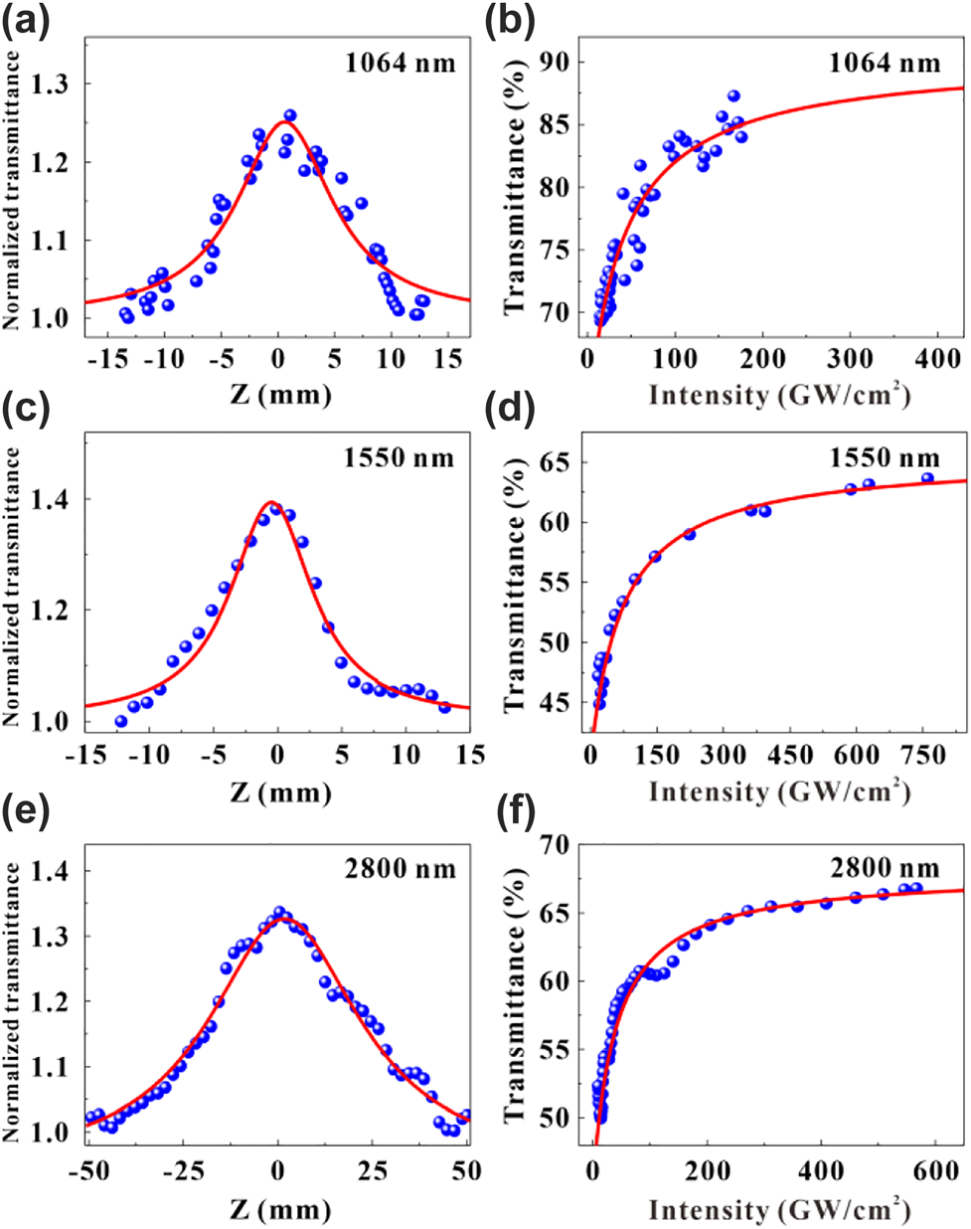
Z-scan characterization. (a), (c) and (e) Nonlinear saturable absorption properties of VO_2_/V_2_O_5_ core–shell heterostructures characterized at 1064 nm, 1550 nm, and 2800 nm, respectively. (b), (d), and (f) Transmittance plotted as a function of input intensity corresponds to 1064 nm, 1550 nm, and 2800 nm, respectively.

The nonlinear optical properties of the VO_2_/V_2_O_5_ core–shell heterostructures were characterized at 1064 nm, 1550 nm, and 2800 nm with the open aperture Z-scan technique, respectively [53]. The pulse duration and repetition rate of the light source used in the experiment were 35 fs and 1 kHz, respectively. Figure 4 shows the experimental results of the Z-scan measurements, showing the saturable absorption of the materials, where the peak of the curves is located at the focus of the beam. The curves of the Z-scan are fitted with a nonlinear transmission function between the light transmittance *T* and the incident light intensity *I*:

(1) $$T=1-\left(\frac{\alpha_{\text{s}}}{1+\frac{I}{I_{\text{sat}}}}+\alpha_{\text{ns}}\right)$$

where *α*_s_ is the modulation depth, *α*_ns_ is the nonsaturable loss, and *I*_sat_ is the saturation intensity [54, 55]. By fitting the experimental results, the modulation depth, saturation intensity and nonsaturable loss have been extracted to be 27%, 42 GW/cm^2^ and 13% at 1064 nm, 23%, 78 GW/cm^2^ and 37% at 1550 nm, and 16.5%, 63.9 GW/cm^2^ and 32% at 2800 nm, respectively. In addition, the VO_2_/V_2_O_5_ heterostructures can withstand laser intensity of at least 200 GW/cm^2^ at 1064 nm, 770 GW/cm^2^ at 1550 nm and 600 GW/cm^2^ at 2800 nm based on the Z-scan experimental results, respectively.

## 3 Results and discussion

**Figure 5:**
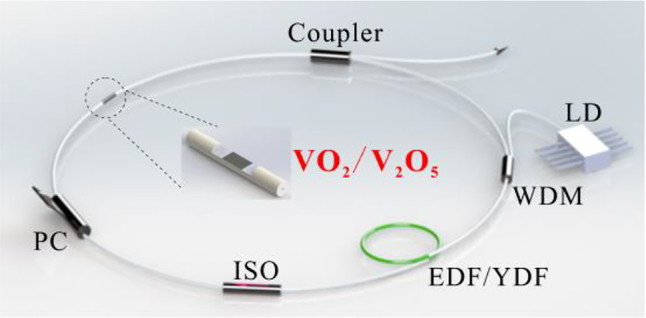
Experimental setup of the 1 μm and 1.5 μm mode-locked fiber laser.

**Figure 6:**
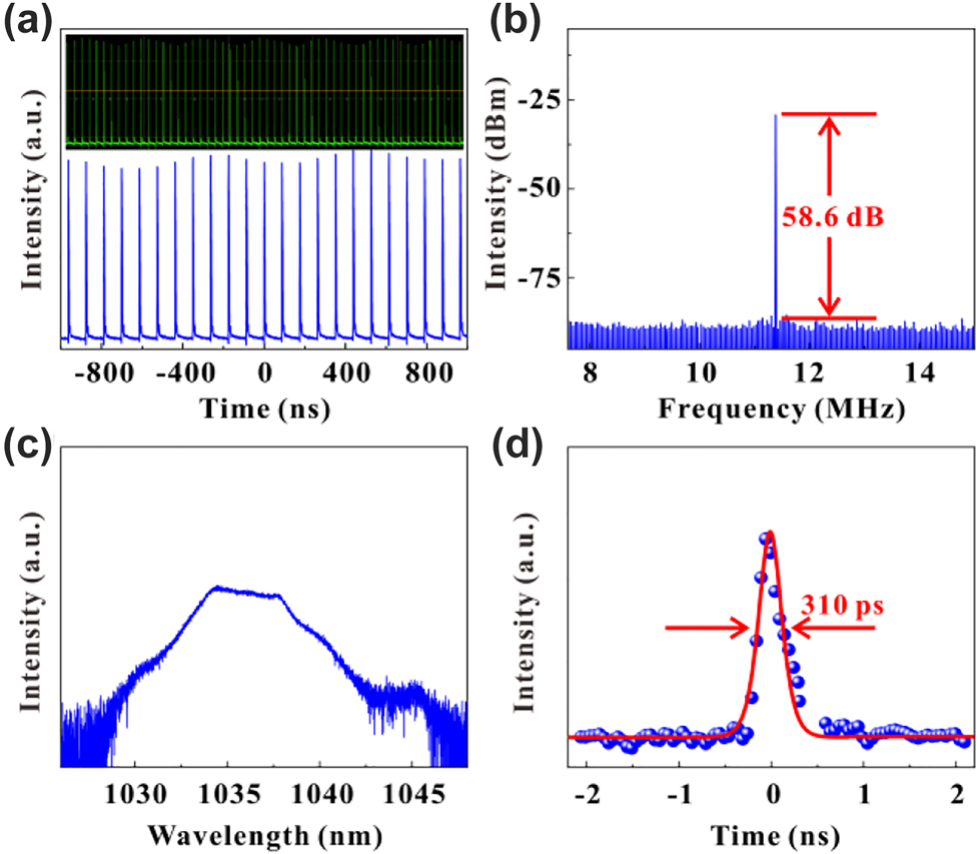
Experimental results of a 1 µm mode-locked fiber laser. (a) The pulse sequence displayed on the oscilloscope. (b) The RF spectrum. (c) Spectral diagram. (d) Single-pulse diagram.

To investigate the nonlinear optical response, the VO_2_/V_2_O_5_ core–shell heterostructures have been introduced into pulsed fiber lasers operating at different wavelengths as nonlinear optical modulators. The laser application of VO_2_/V_2_O_5_ core–shell heterostructures was investigated using a 1 μm Yb-doped gain fiber ring cavity, and the experimental setup is shown in Figure 5. The ring cavity consists of a 980 nm pump source (LD), a 980/1060 wavelength division multiplexer (WDM), a Yb-doped gain fiber, an isolator (ISO), a polarization controller (PC), a D-shaped fiber, a coupler, and a single-mode fiber. The VO_2_/V_2_O_5_ core–shell heterostructures are transferred to the D-shaped fiber to act as the SA. The output of the ring cavity is observed with an oscilloscope (DS09404A) as well as a spectrometer (Ando AQ-6317B). Figure 6a shows a sequence of oscilloscope traces for the mode-locking when the incident light intensity is 320 mW. The RF spectrum is shown in Figure 6b, which shows that the signal-to-noise ratio (SNR) of the output laser is about 58.6 dB with a frequency of 11.4 MHz, indicating the stable performance of the experimentally obtained mode-locked laser. Figure 6c shows the spectrum of the laser with a central wavelength of 1036 nm. The shortest width of the pulse obtained is 310 ps, which matches the hyperbolic secant function fit, as shown in Figure 6d.

**Figure 7:**
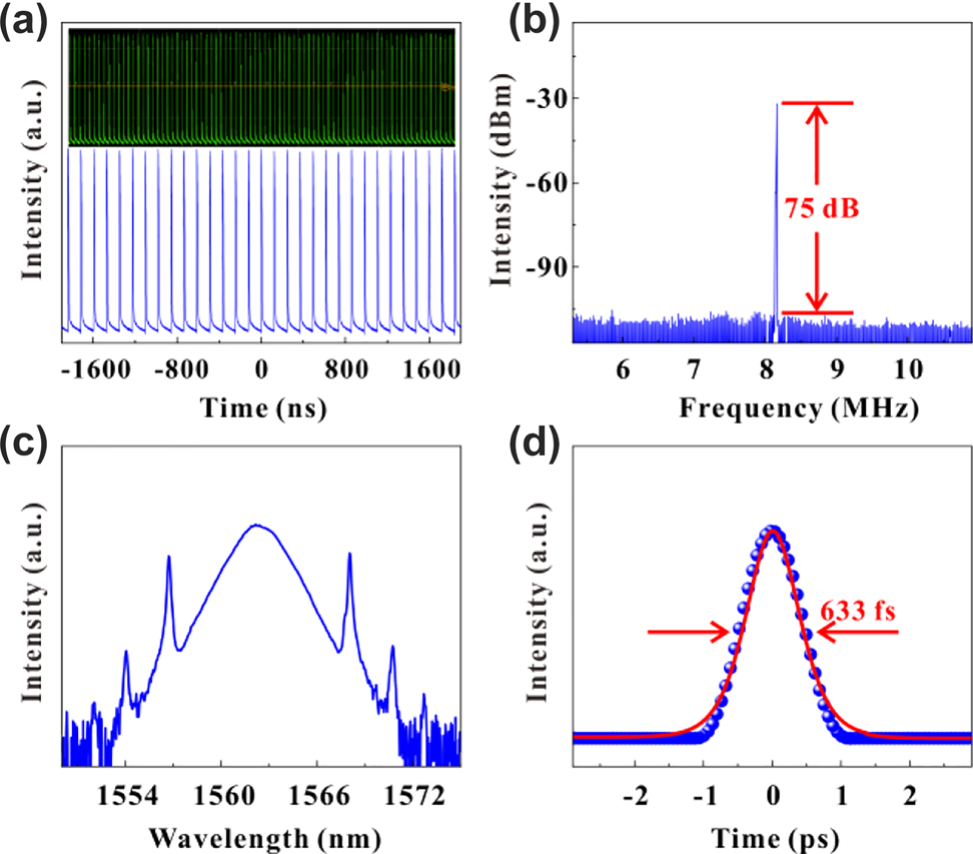
Experimental results of a 1.5 µm mode-locked fiber laser. (a) The pulse sequence displayed on the oscilloscope. (b) The RF spectrum. (c) Spectral diagram. (d) Single-pulse diagram.

To further investigate the nonlinear optical properties of VO_2_/V_2_O_5_ core–shell heterostructures in the near-infrared waveband. The laser application of VO_2_/V_2_O_5_ core–shell heterostructures was investigated using a 1.5 µm Er-doped gain fiber ring cavity, and the experimental setup is shown in Figure 5. The power of the pump source gradually increases from zero, and a stable mode-locking phenomenon appears in the oscilloscope when the power is 140 mW. Figure 7a shows a sequence of oscilloscope traces for the mode-locked pulse when the incident light intensity is 300 mW. The RF spectrum is shown in Figure 7b, which shows that the signal-to-noise ratio of the output laser is about 75 dB with a frequency of 8.1 MHz, indicating the stable performance of the experimentally obtained mode-locked laser. It is worth mentioning that the SNR of the VO_2_/V_2_O_5_ core–shell heterostructures-modulated fiber laser is larger than that of most of the reported low-dimensional materials mode-locked lasers, such as MXene [54], perovskite [56], and BP [57]. Figure 7c shows the spectrum of the laser with a central wavelength of 1562 nm. The single pulse autocorrelation trace is shown in Figure 7d, which matches the hyperbolic secant function fit, and the actual pulse width is 633 fs.

**Figure 8:**
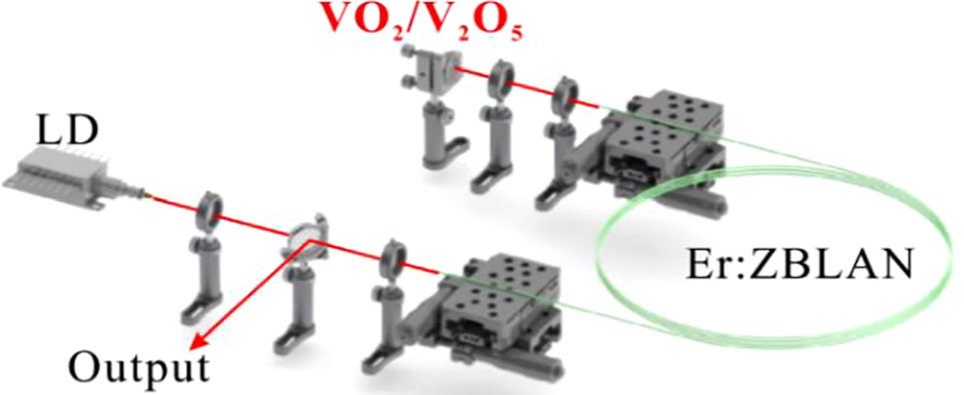
Experimental setup of the 2.8 μm Q-switched fiber laser.

**Figure 9:**
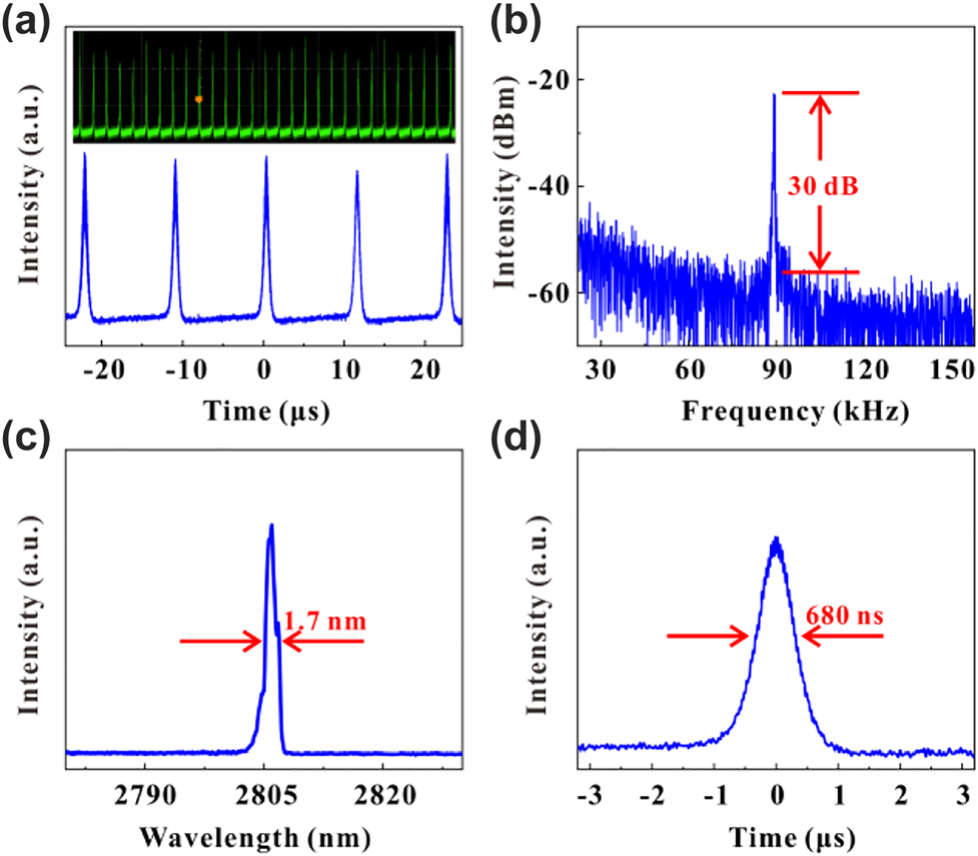
Experimental results of the 2.8 µm Q-switched laser. (a) The pulse sequence displayed on the oscilloscope. (b) The RF spectrum. (c) Spectral diagram. (d) Single-pulse diagram.

We have carried out the nonlinear optics applications of the VO_2_/V_2_O_5_ core–shell heterostructures towards mid-infrared spectral range. The Er-doped fluoride fiber laser experimental setup is shown in Figure 8, where a 976 nm laser diode (LD) is used as the pump source, and an Er: ZBLAN fiber with a core diameter of 15 µm is used as the gain medium. The experimental samples are adhered to the surface of the reflector and the final laser pulses are delivered through the dichroic mirror. The remaining are all lenses, which together form the entire optical system. VO_2_/V_2_O_5_ core–shell heterostructures were used as the SA for the nonlinear modulation of the laser. When the pump power is increased to 1.6 W, the oscilloscope shows a stable Q-switched pulse corresponding to a repetition rate of 60.7 kHz and a pulse width of 984.7 ns. Figure 9a shows a sequence of oscilloscope plots for the Q-switched pulse when the incident light intensity is 2.55 W. The RF spectrum is shown in Figure 9b, which shows that the SNR of the output laser is about 30 dB with a repetition rate of 89 kHz, indicating the stable performance of the experimentally obtained Q-switched laser. Figure 9c shows the spectrum of the laser with a central wavelength of 2.805 μm and a full width at half maximum (FWHM) of 1.71 nm. At the same time, the shortest pulse width obtained is 680 ns, as shown in Figure 9d. The maximum output power of the 2.8 µm Q-switched fiber laser is 0.2 W.

During the experiment, the SA devices can operate at a maximum pump power of 500 mW for the Er-doped and Yb-doped fiber lasers, and we did not observe any damage to the devices. The maximum pump power in the 2.8 µm Q-switched fiber laser can reach 2.55 W, and no damage to the VO_2_/V_2_O_5_ SA devices has been observed. Furthermore, the SA devices with VO_2_/V_2_O_5_ core–shell heterostructures can operate in fiber lasers for more than 4 h with almost no change in laser pulse output. In addition, we did not observe any damage to the VO_2_/V_2_O_5_ SA devices during the whole operation.

**Figure 10:**
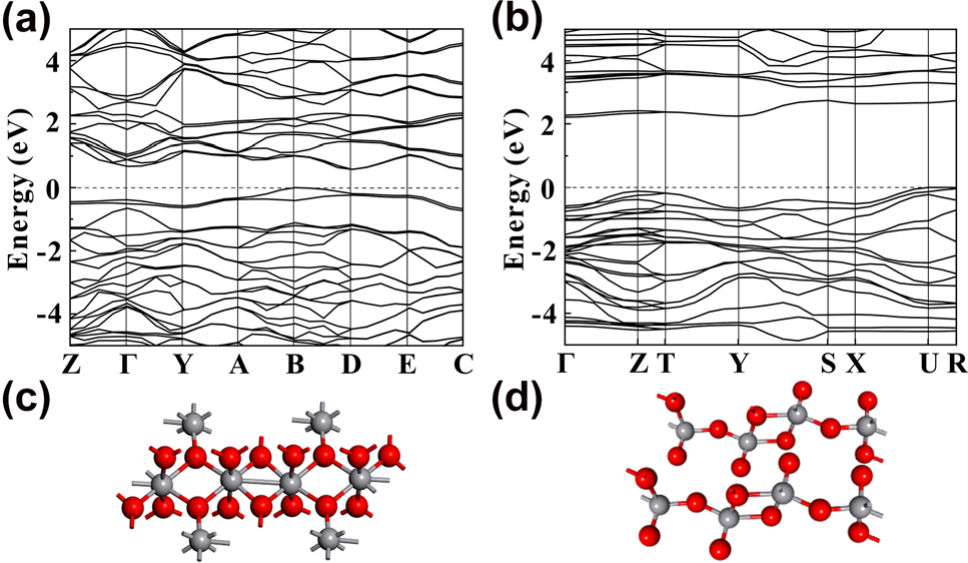
Energy band diagrams and the molecular structure. (a) Energy band diagram of VO_2_. (b) Energy band diagram of V_2_O_5_. (c) Molecular structure of VO_2_. (d) Molecular structure of V_2_O_5_.

**Figure 11:**
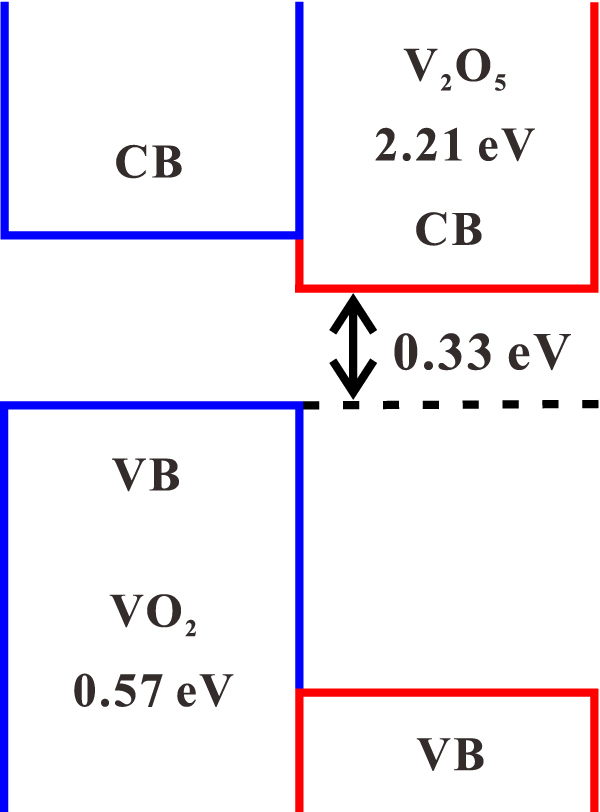
Energy diagrams of VO_2_/V_2_O_5_ core–shell heterostructures.

The molecular structures and the energy band diagrams of VO_2_ and V_2_O_5_ have been studied numerically with first principles method, as shown in Figure 10. The calculated bandgaps for VO_2_ and V_2_O_5_ are 0.57 eV and 2.21 eV, respectively, which are close to the reported 0.6 eV [58] and 2.25 eV [59]. According to the results obtained from the calculations, VO_2_ can absorb light at wavelengths less than about 2.1 µm, while V_2_O_5_ can respond to the light with wavelengths less than about 560 nm. With the consideration, neither VO_2_ nor V_2_O_5_ can respond to light with a wavelength of 2.8 µm. However, according to the reported results, a type II heterostructure can be formed between VO_2_ and V_2_O_5_ [60]. Under the excitation of light, the nonlinear optical absorption can occur between the staggered arrangement of energy bands beyond the intrinsic band gaps of VO_2_ and V_2_O_5_ [43], as shown in Figure 11. The gap between the maximum value of the valence band of VO_2_ and the minimum value of the conduction band of V_2_O_5_ is 0.33 eV, which responds to mid-infrared light with wavelengths shorter than 3.7 μm. As a result, the VO_2_/V_2_O_5_ core–shell heterostructures can act as a nonlinear optical modulator to modulate the Er-doped fluoride fiber laser to deliver mid-infrared Q-switched laser successfully.

The parameters of the Q-switched pulses for different types of SA near the wavelength of 2.8 µm are listed in Table 1. From the reported experimental results, it can be seen that the passively Q-switched fiber laser modulated by VO_2_/V_2_O_5_ core–shell heterostructures can deliver pulses with the relatively short pulse duration, indicating that VO_2_/V_2_O_5_ core–shell heterostructures have excellent nonlinear optical modulation performance in the mid-infrared regime.

**Table 1:** Laser pulse parameters for Q-switching by different types of SA near 2.8 µm wavelength.

SA	Wavelength (μm)	Pulse period (μs)	SNR (dB)	Modulation depth (%)	Saturation intensity	Ref.
MXene-Ti_3_C_2_T_x_	2.8	1.04	–	33.20	0.043 GW/cm^2^	[11]
MXene-Ti_3_C_2_T_x_	2.73	0.814	–	1.75	45.5 kW/cm^2^	[12]
Carbon nanotube	2.87	1.21	50	16.5	1.66 MW/cm^2^	[13]
MoS_2_	2.8	0.806	40	5	–	[14]
BP	2.8	1.18	35	15	–	[15]
Graphene	2.78	2.9	30	–	–	[17]
Bi_2_Te_3_	3	1.37	37.4	51.3	2.12 MW/cm^2^	[20]
Antimonene	2.87	1.74	34.7	10.5	0.26 GW/cm^2^	[22]
Cu_2−x_S	2.77	0.75	30	–	–	[55]
Antimonene	3.47	3.7	39.9	8	–	[61]
InSe	2.77	1.2	42.4	12	–	[62]
Doped ZnO	2.79	0.56	37	–	–	[63]
	3.46	1.78	30			
VO_2_/V_2_O_5_	2.8	0.68	30	16.5	63.9 GW/cm^2^	This work

## 4 Conclusions

In summary, we have experimentally investigated the nonlinear optical properties of VO_2_/V_2_O_5_ core–shell heterostructures and revealed the broadband nonlinear optical response of VO_2_/V_2_O_5_ core–shell heterostructures toward the mid-infrared regime. The core–shell heterostructures have been introduced into pulsed fiber lasers operating at different wavelengths as nonlinear optical modulators. The shortest pulse widths of 310 ps, 633 fs, and 680 ns from different fiber lasers have been demonstrated at 1064 nm, 1550 nm, and 2800 nm wavebands, respectively. This work indicates that the VO_2_/V_2_O_5_ core–shell heterostructures can exhibit broadband nonlinear optical responses and may lay the foundation for their application in broadband optoelectronic devices, especially towards mid-infrared spectral range.
